# An Enhanced Absolute Eddy Current Probe for Surface Cracks Detection at High Temperatures

**DOI:** 10.3390/s26134056

**Published:** 2026-06-26

**Authors:** Zhiying Liu, Wenze Shi, Chao Lu, Tuan Zhu, Hongyu Sun, Zhonghao Luo, Gongpeng Yang, Yiping Liang

**Affiliations:** 1 Key Laboratory of Non-Destructive Testing, Ministry of Education, Nanchang Hangkong University, Nanchang 330063, China; liuzhiying20231@163.com (Z.L.); zhutuan0528@outlook.com (T.Z.); 18270649580@163.com (Z.L.); 2 School of Physical Science and Engineering, Beijing Jiaotong University, Beijing 100044, China; hysun@bjtu.edu.cn; 3 Intelligent Non-Destructive Testing Center, Taihang National Laboratory, Nanchang 330096, China; yanggongpeng@buaa.edu.cn (G.Y.); lypddup@163.com (Y.L.)

**Keywords:** high-temperature eddy current testing, nickel-based alloy, optimal design, water-cooled sensor, surface cracks

## Abstract

Non-destructive evaluation of surface cracks in Inconel 718 nickel-based alloys operating at high temperatures is crucial for monitoring aero-engine hot-section components. Conventional eddy current testing is often constrained by thermal core degradation and low signal-to-noise ratios, struggling to meet detection requirements in such extreme environments. To address this, this study proposes an optimized absolute probe integrated with an efficient water-cooling system. A multi-physics finite element model was developed to optimize the probe design, focusing on key parameters such as excitation frequency and the geometric dimensions of the coil and ferrite core. Experimental results demonstrate that the optimized probe significantly enhances detection sensitivity over conventional models. Specifically, the peak amplitude increased by 76.2% and the signal-to-noise improved by nearly 10 dB for a 0.3 mm-deep crack. In practical applications, the probe achieves high-sensitivity detection of a 0.3 mm-deep crack at 500 °C. At 600 °C, it reliably detects a 0.5 mm-deep crack with a coefficient of variation not exceeding 3.5% and it retains detection capabilities even at 650 °C. Therefore, this sensor design strategy proves to be a highly viable method for non-destructive evaluation in extreme industrial thermal environments.

## 1. Introduction

Inconel 718 nickel-based alloys are vital for aero-engine hot-section components due to their exceptional high-temperature strength and fatigue resistance [1]. However, prolonged service under extreme conditions often leads to surface microcracks, compromising component integrity and equipment safety [2]. Consequently, developing reliable in-service monitoring technologies is critical for predictive maintenance. Conventional eddy current testing (ECT) serves as an effective solution, offering non-contact measurement, high sensitivity to surface defects, and couplant-free operation, making it ideal for harsh, high-temperature industrial environments [3].

Despite the numerous advantages of ECT, such as its non-contact nature, conventional applications at high temperatures face significant challenges, including thermal degradation of probe materials, signal drift, and temperature-induced electromagnetic variations. To overcome these bottlenecks, numerous researchers have conducted extensive studies over the past decade. For instance, Klümper et al. [4] developed an eddy current sensor capable of withstanding 500 °C, achieving in-service nondestructive monitoring of microstructural evolution by translating harmonic analysis into real-time electrical signals. Furthermore, to improve micro-crack detection, continuous innovations have been made in probe structures and theoretical modeling, such as exact analytical models proposed for rectangular side-by-side probes to guide sensor optimization [5].

However, the reliability of such multi-coil or complex structures is often poor under severe thermal perturbations. Therefore, for practical high-temperature inspection, research has shifted toward more robust probe designs and multi-technology fusion. Crivellaro et al. [6] developed a customized system equipped with active water-cooling and thermal insulation, enabling stable operation at 340 °C for detecting 0.4~0.5 mm defects. Building on this, Santos et al. [7] integrated ultrasonic and eddy current technologies to construct an automated inspection system capable of identifying 0.3 mm weld cracks and 2.5 mm lack-of-fusion defects within the 200~300 °C range, verifying the effectiveness of multi-technology integration.

Regarding inspection scope and efficiency, emerging multi-physics coupling methods have demonstrated tremendous potential. Jiang et al. [8] coupled electromagnetic induction heating with infrared thermography to enable simultaneous surface imaging, quantitatively evaluating cracks up to 4 mm deep. Addressing the demand for rapid inspection, Li et al. [9] developed a dynamic scanning system based on multi-physics structured eddy current thermography, significantly improving efficiency for large-area testing. Despite these advancements, current systems for detecting sub-millimeter (0.3~0.5 mm) micro-cracks are largely limited to the 300~350 °C range. Maintaining high detection sensitivity at temperatures above 400 °C under severe thermal interference remains an urgent engineering bottleneck.

To address the above issues, this study combines field-circuit coupled finite element optimization and comprehensive thermal management design. This significantly improves the high-temperature tolerance and detection precision of absolute eddy current probes. The study improves the room-temperature baseline sensitivity to 0.1 mm and effectively extends this capability into severe high-temperature conditions. For the Inconel 718 superalloy, the developed probe accurately detects 0.3 mm micro-cracks at 450 °C, surpassing existing 300 °C-level systems. Crucially, it also maintains stable detection of 0.5 mm cracks under extreme conditions up to 650 °C. These results significantly improve crack detection capabilities at extreme temperatures and provide an experimental basis for understanding crack evolution in severe service environments.

## 2. Finite Element Model for ECT of Superalloys

### 2.1. Mechanism Analysis of High-Temperature ECT

Absolute eddy current probes are widely used for the high-sensitivity detection of surface fatigue cracks due to their compact structure and lack of directional blind spots. When energized by an alternating current of frequency *f*, the base self-impedance of a probe coil consists of its resistance and inductive reactance. In high-temperature environments, the coil’s inductance is minimally affected by temperature and can be treated as a constant L_0_ due to the physical properties of pure copper. This can be expressed as:

(1) $$Z_{c}=R_{c}+jwL_{0}$$

Conversely, continuous heating causes a surge in the resistance of the pure copper coil, which increases sharply and linearly with the rising ambient temperature *T*, expressed as:

(2) $$R_{c}(T)=R_{0}[1+\alpha_{c}(T-T_{0})]$$

where *R*_0_ is the initial resistance at the reference temperature *T*_0_, and α_c_ is the temperature coefficient of resistance for copper.

The resistance surge-induced thermal disturbance introduces a strong baseline drift in detection signals. To suppress this effect, a water-cooling device is employed to alter the heat transfer boundary conditions. Through fluid convective cooling, the coil and magnetic core are maintained at a low quasi-steady-state temperature. This achieves a physical decoupling between the probe self-impedance and the external environment, yielding the following approximation:

(3) $$Z_{c}(T_{c})\approx Z_{c}(T)$$

where *T_c_* is the quasi-steady-state operating temperature of the probe under water cooling, and *T* is the external high-temperature ambient temperature.

Water-cooling decoupling suppresses the probe’s thermal drift. This isolates the actual physical response of the tested material. Therefore, the impedance drift stems directly from the high-temperature degradation of Inconel 718. Elevated temperatures increase lattice vibrations and electron-phonon scattering. This leads to a nonlinear decrease in electrical conductivity. As a result, the alternating magnetic field’s skin depth expands:

(4) $$\delta (T)=\frac{1}{\sqrt{\pi f\mu \sigma (T)}}$$

Surface eddy current diffusion shifts the reflection impedance. This degradation creates a downward baseline drift in the real scan profile. As a result, the probe output nonlinearly combines thermal drift and crack distortions. Temperature gradients and thermal expansion further result low-frequency drift. As temperatures approach 600 °C, this drift spikes. It severely compresses the signal amplitude and can completely hide crack peaks. Since hardware isolation is inadequate at these extreme temperatures, baseline subtraction is essential to extract weak crack signals.

### 2.2. Three-Dimensional Finite Element Modeling

The 3D finite element model for eddy current testing established in this study mainly consists of an Inconel 718 superalloy plate, probe coils, a ferrite core, and an air domain. The geometric dimensions of the alloy plate are set to 50 mm in length, 50 mm in width, and 2 mm in thickness. A rectangular artificial notch, used to simulate a defect, is located at the center of the plate with a length of 8 mm, a width of 0.2 mm, and a depth of 0.2 mm.

**Figure 1:**
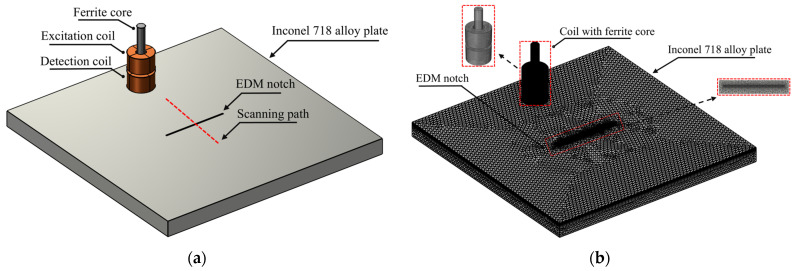
Finite element simulation of the absolute eddy current probe: (**a**) Configuration of the 3D simulation model; (**b**) Meshing strategy of the complete model with magnified views.

The simulation models an induced absolute probe with two vertical coaxial coils: the upper one is the excitation coil, and the lower one is the detection coil. Both coils share identical parameters: a 0.6 mm inner diameter, a 0.96 mm outer diameter, a 2 mm height, and 200 turns. A manganese-zinc ferrite core (1 mm diameter, 8 mm height) is inserted to enhance magnetic coupling. The scanning path in the simulation is set such that the probe scans linearly perpendicular to the length of the notch. The excitation source is an alternating voltage with an amplitude of 8 V and a frequency of 50 kHz. Figure 1a shows the constructed 3D model.

The 3D finite element model was implemented in COMSOL Multiphysics (version 6.3, COMSOL AB, Stockholm, Sweden). The electromagnetic field source was modeled as a distributed current. The Homogeneous Multi-Turn Coil feature was utilized for this purpose. This feature distributed the current density uniformly across the coil cross-sections. To truncate computational boundaries efficiently, the airspace was divided into nested near-field and far-field domains. The inner near-field air box surrounded the probe and the defect zone, which was 100 mm long, 100 mm wide, and 32 mm high. The outer far-field air box enclosed the entire physical model measuring 180 mm long, 180 mm wide, and 40 mm high. Furthermore, the manganese-zinc ferrite core was explicitly included as a solid entity within this calculation region. The magnetic field interactions of the core were fully accounted for during the simulation.

The eddy current model relies on Maxwell’s equations [10]. A frequency-domain solver is used to efficiently compute the time-harmonic electromagnetic fields. The simulation uses the Magnetic and Electric Fields interface in the AC/DC module, applying Ampere’s Law to the entire domain. Setting a magnetic insulation boundary (**A** = 0) at the air domain edges provides a homogeneous Dirichlet condition [11].

The physical properties of each computational domain determine the eddy current distribution [12]. Several simplifying assumptions were made for the numerical model. All solid materials are assumed to be isotropic and homogeneous. Displacement currents are neglected due to the low excitation frequency. Thus, the simulation operates under the quasi-static electromagnetic approximation. The ferrite core maintains a constant relative magnetic permeability. Furthermore, the water-cooling system protects the absolute probe during high-temperature testing. The material properties of the probe are therefore assumed to remain thermally stable. Consequently, thermal drift effects on the probe itself are neglected. The material properties are characterized by electrical conductivity and relative permeability. Table 1 lists the specific values.

Spatial discretization of the geometric model into a mesh is required before solving. Therefore, a localized hybrid meshing strategy was adopted to balance efficiency and accuracy. Free tetrahedral elements were used for the bulk domains, including the air, core, and coils. Refined triangular elements were applied to the surfaces. Crucially, six layers of prismatic boundary layer elements were used on the Inconel 718 specimen surface and notch boundaries. These elements accurately captured the skin effect. For the final optimized configuration, the total element count fluctuates around 1,236,500 and the final mesh setup is illustrated in Figure 1b.

**Figure 2:**
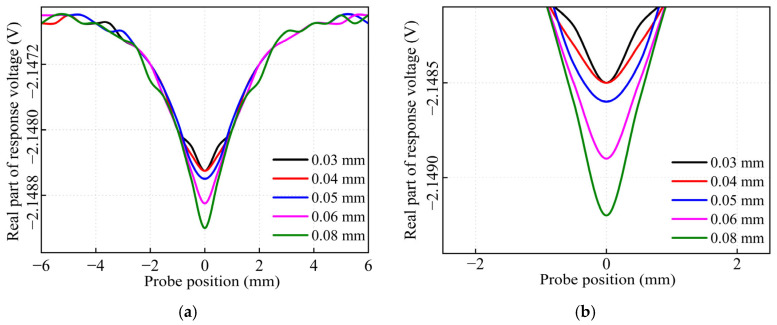
Comparison of real part of response voltage at different grid sizes. (**a**) Overall response curves; (**b**) Local magnification at the defect center.

To evaluate the effect of spatial discretization on the simulation accuracy, a mesh convergence study was conducted by varying the element size in the defect region from 0.08 mm to 0.03 mm. The calculated response voltage curves are shown in Figure 2a, with a close-up view at the defect center provided in Figure 2b. The valley voltage stabilizes as the mesh is refined. Table 2 summarizes the calculated voltages and corresponding relative errors based on the finest 0.03 mm mesh. Because the 0.05 mm mesh yields a negligible relative error of only 0.0093%, it was selected for the simulations to optimize both precision and computational efficiency.

**Figure 3:**
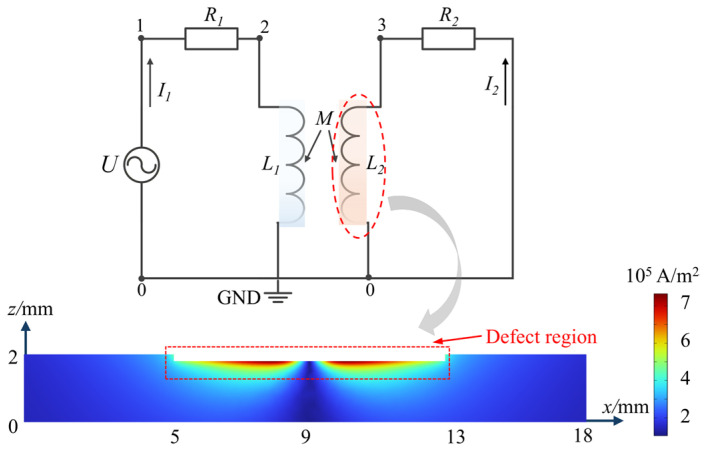
System modeling and simulation analysis: the equivalent circuit model and the corresponding surface current density distribution. The red dashed ellipse highlights the detection coil *L*_2_, and the gray arrow indicates the electromagnetic coupling between detection coil L_2_ and the eddy current field.

Figure 3 shows the complete circuit schematic and the corresponding eddy current density distribution. The circuit comprises an 8 V AC voltage source, 50 Ω resistors R_1_ and R_2_, capacitors, and external coupling modules. The ‘External I vs. U’ module inductively couples excitation coil L_1_ and sensing coil L_2_. As the probe scans, surface defects perturb the specimen’s electromagnetic properties. This perturbation creates measurable voltage fluctuations at Node 3. Furthermore, the induced current density peaks at the surface and decays exponentially with depth [13]. This skin effect grants the probe high sensitivity to near-surface defects. Equation (4) defines the standard penetration depth δ, where the density drops to 1/e of its surface maximum.

## 3. Impact of Inspection Parameters on Electromagnetic Response

Optimal matching of probe parameters is critical for achieving high-sensitivity crack detection at high temperatures. To ensure theoretical rigor, we mathematically formulate the optimization problem. The design parameters include the excitation frequency (*f*), coil turns (*N*), core height (*h*), core diameter (*d*), and coil spatial spacing (*s*). The optimization criterion requires the maximization of defect detection sensitivity. Specifically, the real part of the response voltage variation (Δ*V*_1_) serves as the objective function. The optimization mathematical model is defined as follows:

(5) $$\begin{array}{l} \max\Delta V_{1}(x)\\ \mathrm{subject}\;\mathrm{to}\;x={\left[f,N,h,d,s\right]}^{T} \end{array}$$

where x denotes the design parameter vector.

The parameter bounds of this optimization problem are determined by the structural dimensions of the probe, which take discrete values. The corresponding objective function values are subsequently evaluated using finite element simulations.

To solve this non-linear optimization problem effectively, a step-by-step sequential evaluation is adopted in this study. First, the excitation frequency (*f*) is determined based on the skin depth requirement of the Inconel 718 specimen. Under this fixed frequency baseline, the coil turns are adjusted to analyze the parameter sensitivity [14]. Subsequently, the core diameter and core height are evaluated sequentially. Compared to an orthogonal experimental design, this step-by-step parametric scan avoids physically invalid parameter combinations [15]. It also clarifies the independent impact of each structural variable on the sensor response [14]. This individual sensitivity analysis is essential for ensuring design robustness under thermal environments.

### 3.1. Optimization for Excitation Frequency

**Figure 4:**
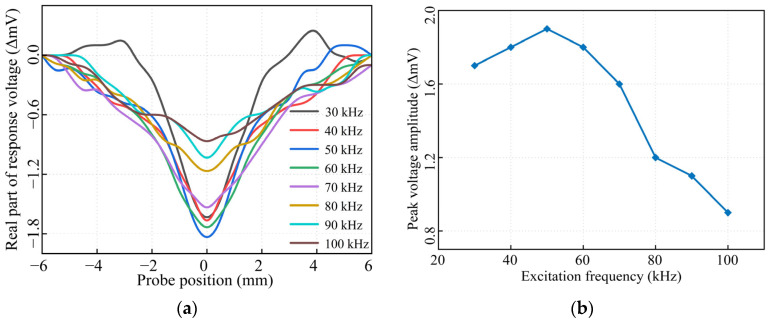
Effect of excitation frequency on response voltage: (**a**) Real voltage variation (Δ*V*_1_) versus probe position; (**b**) Peak amplitude of *ΔV*_1_ versus excitation frequency.

In the simulation, the scanning path of the probe was perpendicular to the defect, and the excitation voltage was maintained at 8 V. The excitation frequency ranged from 30 kHz to 100 kHz with a step size of 10 kHz. To eliminate discretization-induced variations across this frequency range, a standardized mesh configuration was implemented. The first layer thickness of the boundary mesh was fixed based on the skin depth at 100 kHz to ensure sufficient spatial resolution. Meanwhile, the total boundary domain thickness was set to three times the skin depth at 30 kHz to fully contain the electromagnetic field penetration. The real part of the response voltage variation at the detection coil was extracted as the defect signal. Figure 4 displays the calculated response curves and the corresponding peak amplitudes. The voltage curves maintain a symmetric V-shaped profile along the scanning line. At the defect center, the voltage variation amplitude increases from 1.7 mV at 30 kHz to a maximum of 1.9 mV at 50 kHz. Beyond 50 kHz, the variation amplitude decreases continuously, dropping to 0.9 mV at 100 kHz. This trend confirms that 50 kHz balances the eddy current density and the penetration capability within the Inconel 718 specimen, serving as the proper excitation frequency.

### 3.2. Impact of Coil Turns

**Figure 5:**
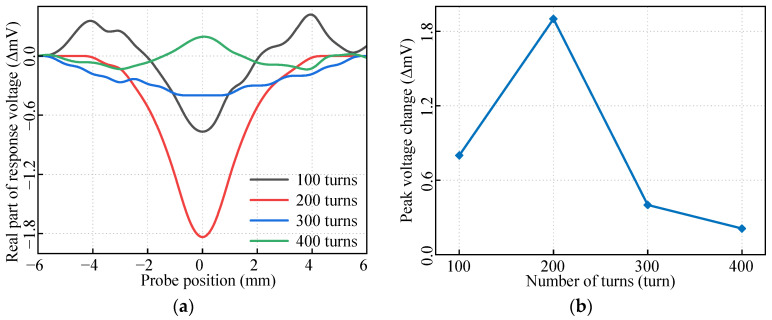
Influence of coil turns on response voltage: (**a**) Real voltage variation (Δ*V*_1_) versus probe position; (**b**) Peak amplitude of Δ*V*_1_ versus the number of turns.

The number of coil’s turns directly determines the electromagnetic coupling strength, sensitivity, and signal-to-noise ratio of the probe [16]. Therefore, this section optimizes the number of turns as a single variable. In the simulation, the test material is set as Inconel 718 alloy. A rectangular defect measuring 8 mm long, 0.2 mm wide, and 0.2 mm deep is located on the surface. The detection coil has a fixed height of 2 mm and an inner diameter of 0.6 mm. The excitation voltage is kept at 8 V. The excitation frequency is set to the previously optimized 50 kHz. The number of turns ranges from 100 to 400 with a step size of 100. The voltage of the detection coil is extracted. The simulation results are shown in Figure 5.

As shown in Figure 5a, the scanning signals for various coil turns all peak exactly at the defect center. However, the peak voltage exhibits a non-linear response to the turn count (Figure 5b). From 100 to 200 turns, the magnetic field strengthens linearly. Beyond 200 turns, a sharp rise in coil resistance drastically reduces the excitation current, negating the benefits of additional turns and weakening the overall magnetic field [17]. Since the signal reaches its maximum (about 1.8 mV) at 200 turns, we selected this as the proper parameter.

### 3.3. Optimization of Core Height

**Figure 6:**
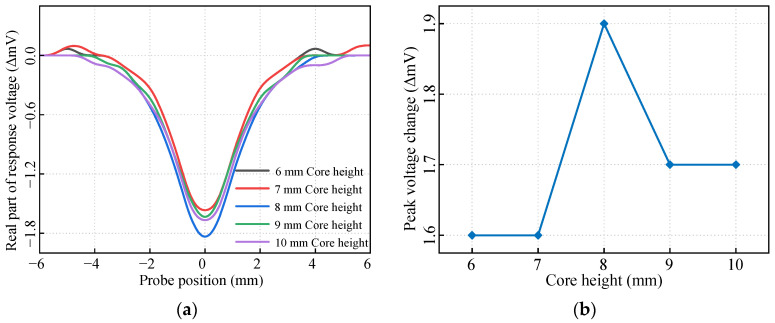
Influence of core height on response voltage: (**a**) Real voltage variation (Δ*V*_1_) versus probe position; (**b**) Peak amplitude of Δ*V*_1_ versus core height.

Based on the previous optimizations, the excitation frequency is fixed at 50 kHz and the number of turns at 200. The coil wire diameter is set to 0.08 mm. Under these parameters, the effect of the core height on the detection signal is investigated. The simulation results are shown in Figure 6. As Figure 6b illustrates, the core height has no significant impact on the signal amplitude. Therefore, a height of 8 mm is finally selected for the ferrite core.

### 3.4. Effect of Core Diameter

**Figure 7:**
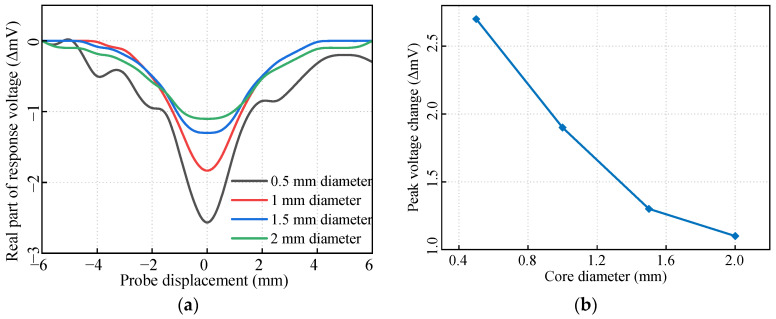
Influence of core diameter on response voltage: (**a**) Real voltage variation (Δ*V*_1_) versus probe position; (**b**) Peak amplitude of Δ*V*_1_ versus core diameter.

Next, we optimized the ferrite core diameter. Other parameters were set to their previously determined optimal values. Figure 7 shows the voltage variations of the detection coil. As shown in Figure 7b, the defect signal amplitude exhibits a clear linear decreasing trend as the core diameter increases. Theoretically, a smaller diameter yields a stronger signal response. However, ferrite cores with a diameter of less than 1 mm face commercial availability and manufacturing limitations. Considering both detection sensitivity and practical engineering feasibility, a core diameter of 1 mm was finally selected.

### 3.5. Optimization of Coil Spatial Configuration

**Figure 8:**
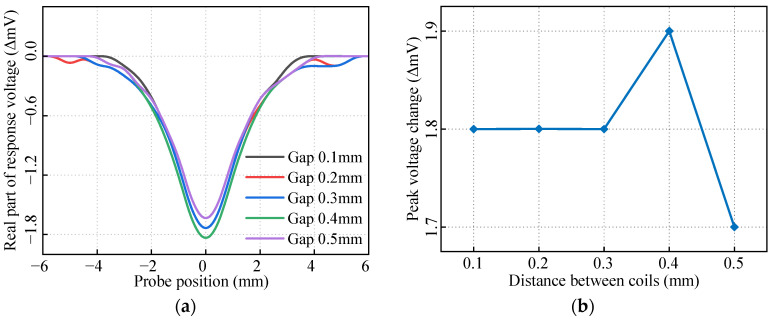
Influence of coil gap on response voltage: (**a**) Real voltage variation (Δ*V*_1_) versus probe position; (**b**) Peak amplitude of Δ*V*_1_ versus the distance between coils.

Rational coils’ spacing can effectively suppress electromagnetic crosstalk and significantly improve detection sensitivity [18]. In this section, we optimized the coil spacing while keeping the magnetic core and other parameters at their optimal values. The spacing was increased from 0.1 mm to 0.5 mm with a step size of 0.1 mm. The extracted voltage variations of the detection coil is shown in Figure 8. The simulation results indicate that the detection signal reaches its peak when the spacing is 0.4 mm. Therefore, 0.4 mm was selected as the proper coil spacing for subsequent experiments.

**Figure 9:**
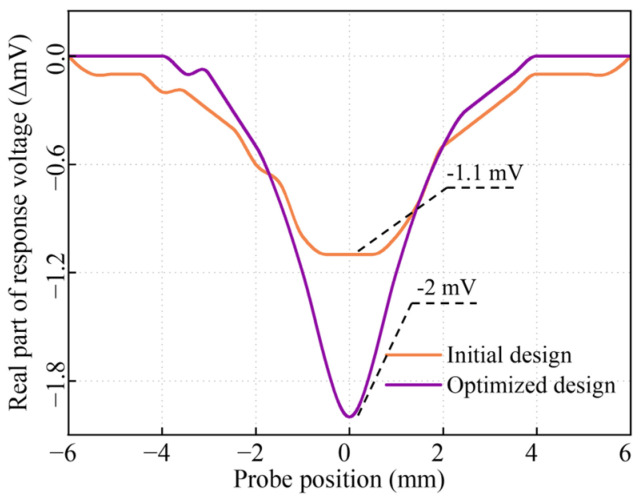
Comparison of the real part of the response voltage between the initial and optimized probe designs scanning a 0.2 mm-deep surface crack.

Simulation analysis yielded the following optimal parameters for the high-temperature absolute eddy current probe: 50 kHz excitation frequency, 200 turns, 0.4 mm coil spacing, 8 mm core height, and 1 mm core diameter. We compared this optimized probe against an initial design (300 turns, 10 mm core height, 2 mm core diameter). As shown in Figure 9, the optimized design doubles the signal amplitude, significantly enhancing detection sensitivity.

### 3.6. Lift-Off Sensitivity Analysis

An equivalent transformer model is employed for investigating thermally induced lift-off variations under extreme environmental test conditions. In this model, the parallel probe coils and the Inconel 718 specimen represent the primary and secondary circuits, respectively. The absolute probe’s total equivalent impedance *Z* is expressed as:

(6) $$Z=\left(R_{1}+\frac{\omega^{2}M^{2}R_{2}}{R_{2}^{2}+{\left(\omega L_{2}\right)}^{2}}\right)+j\left(\omega L_{1}-\frac{\omega^{3}M^{2}L_{2}}{R_{2}^{2}+{\left(\omega L_{2}\right)}^{2}}\right)$$

where *R*_1_ and *L*_1_ are the inherent resistance and self-inductance of the primary coils; *R*_2_ and *L*_2_ are the equivalent resistance and inductance of the eddy current field in the specimen; and M is the mutual inductance. In high-temperature environments, thermal expansion causes micro-variations in the lift-off distance, which exponentially modulates *M*.

During high-temperature testing, micro-variations in lift-off exponentially modulate the mutual inductance *M*, imposing obvious baseline drift and arbitrary phase trajectories on original eddy current signal. However, the derived equations indicate that lift-off and crack perturbations possess distinct, orthogonal phase characteristics in the complex plane. Consequently, applying a phase rotation completely decouples the massive lift-off drift from the primary defect channel. This orthogonal separation ensures that the designated detection feature remains highly robust against lift-off interference amid thermal noise.

To quantitatively validate the theoretical phase-separation mechanism, a numerical sensitivity analysis was executed utilizing the finite element model. The simulation was conducted at a constant thermal condition of 600 °C using a defect-free Inconel 718 specimen to completely isolate the lift-off perturbation. The probe lift-off distance was swept sweepingly from 0.5 mm to 1.5 mm and the step size is 0.25 mm. Following standard eddy current signal processing methods, a phase rotation calibration with an optimized angle was applied to the raw complex voltage outputs to project the dominant lift-off trajectory strictly along the imaginary axis.

**Figure 10:**
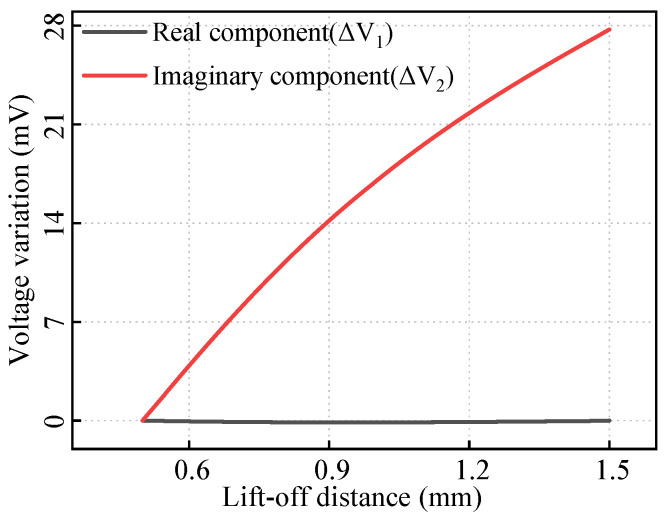
Quantitative variation of the background-subtracted real (Δ*V*_1_) and imaginary (Δ*V*_2_) voltage components as a function of the probe lift-off distance after applying a phase rotation calibration (Simulated at 600 °C on a defect-free Inconel 718 specimen).

Figure 10 illustrates the baseline-corrected results baselined at 0.5 mm lift-off. The calibrated data reveal that the rotated imaginary component (Δ*V*_2_) captures the dominant lift-off interference, exhibiting an increase of approximately 28 mV. Conversely, the rotated real component (Δ*V*_1_) remains stable near 0 mV across the tested lift-off range.

This high consistency between the simulated voltage trends and the circuit model predictions validates the theoretical derivation. The results demonstrate that after appropriate phase separation, the real component of the response voltage (Δ*V*_1_) achieves near-complete immunity to severe lift-off variations, thereby serving as an optimal, noise-immune feature for accurate crack characterization.

### 3.7. Finite Element Simulation of Probe Responses to Varying Crack Depths

To evaluate the probe’s detection limit for shallow defects, finite element simulations were conducted at ambient temperature for cracks with depths of 0.1 mm, 0.2 mm and 0.3 mm.

**Figure 11:**
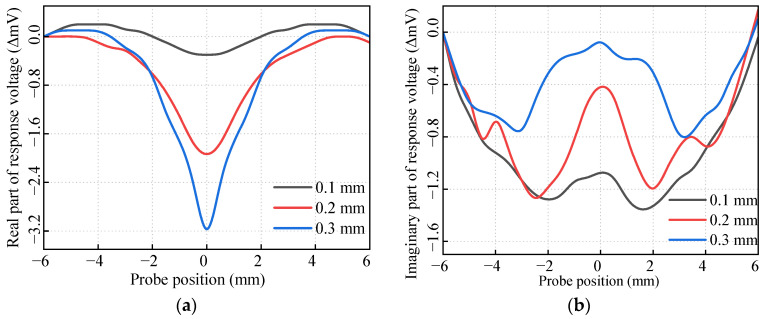
Response characteristics of the sensor for different crack depths: (**a**) Real voltage variation (Δ*V*_1_) versus probe position; (**b**) Imaginary voltage variation (Δ*V*_2_) versus probe position.

As shown in Figure 11a, even for the 0.1 mm-deep crack, the real part of the response voltage still exhibits a clear V-shaped signal feature at the crack center. The probe configuration was optimized based on a 0.2 mm-deep crack benchmark to meet the technical requirement of detecting early fatigue cracks in high-temperature environments. As shown in Figure 11a, the real part of the response voltage variation exhibits a symmetric profile centered. At the crack center, the peak voltage variation values for 0.1 mm, 0.2 mm, and 0.3 mm-deep cracks are 0.3 mV, 2.0 mV, and 3.6 mV. This monotonic increase confirms a high correlation between the peak response and the defect depth. Theoretically, a change in crack size or temperature will alter the eddy current distribution and change the optimal parameters. However, the current configuration is considered sufficient for detecting cracks within the targeted range of this study.

### 3.8. Finite Element Simulation Analysis of Temperature Effects on Defect Signals

Thermal drift of probe impedance critically limits high-temperature testing sensitivity. To investigate its effect on Inconel 718 defect signals, a finite element model incorporating temperature-dependent properties is established to replicate experiments.

Table 3 defines the conductivities and Young’s moduli of copper and Inconel 718 [19,20]. To highlight the dominant electromagnetic mechanisms, other parameters are excluded. The relative permeabilities of copper and Inconel 718 remain near 1, and quasistatic dielectric variations are negligible. Additionally, forced water-cooling keeps the ferrite core below its curie point. While residual thermal gradients may cause minor coil expansion and permeability fluctuations, they only induce a slow background drift in the eddy current signal. Since baseline subtraction eliminates this drift during signal processing, the core’s permeability is modeled as constant. Meanwhile, Equation (2) is introduced into the equivalent circuit for the coil’s resistance. This section evaluates thermal drift by analyzing the real-part voltage variation of the detection coil.

**Figure 12:**
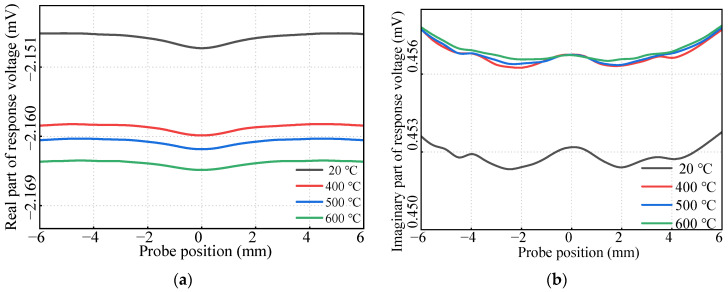
Response characteristics of the sensor at different temperatures: (**a**) Real part of the response voltage versus probe position; (**b**) Imaginary part of the response voltage versus probe position.

Figure 12 shows the simulated scanning responses for a 0.2 mm-deep crack from 20 °C to 600 °C. The results indicate a clear difference in thermal stability between the real and imaginary parts. In Figure 12a, the real voltage baseline shifts uniformly due to the drop in the alloy’s conductivity, yet the central defect signal remains consistent and clear. Notably, as the temperature increases, the amplitude of the real part of the defect response gradually decreases from 2 mV to 1.1 mV. In contrast, the defect signal in the imaginary part (Figure 12b) is gradually drowned out by noise as the temperature rises, making it difficult to distinguish.

## 4. Experimental Verifications of ECT for Nickel-Based Alloys

Based on the previously determined optimal probe parameters, this chapter builds a high-temperature eddy current testing system. Actual inspections were conducted on defects in Inconel 718 alloy plates. By comparing the experimental and simulation results, the actual performance of the optimized probe was verified. This further proves the feasibility and effectiveness of the system for detecting alloy cracks in high-temperature environments.

### 4.1. High-Temperature Testing System and Specimen

**Figure 13:**
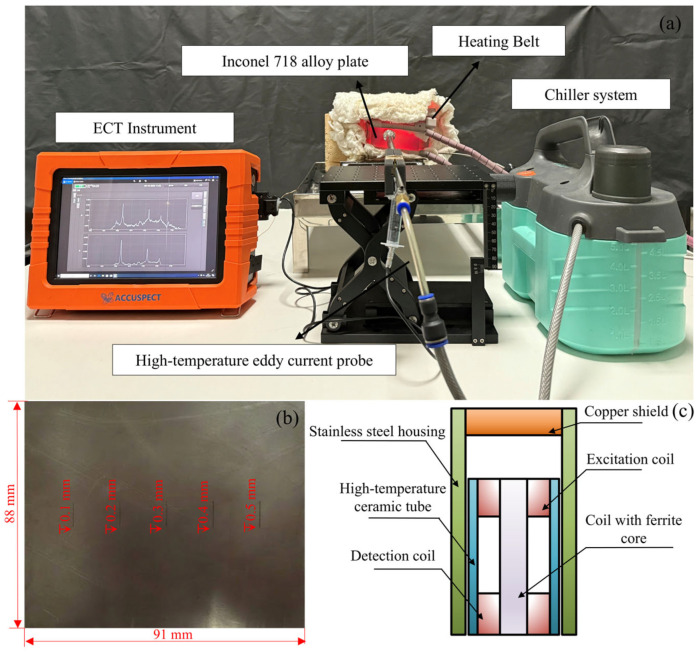
High-temperature eddy current testing system: (**a**) High-temperature experimental setup; (**b**) Inconel 718 alloy plate (2 mm thick) with five artificial cracks (0.1~0.5 mm depth) spaced at 15 mm intervals from left to right; (**c**) Schematic diagram of the absolute eddy current probe. The design features robust materials and an active cooling system for precise crack detection in extreme thermal environments.

A complete high-temperature eddy current testing platform was established showed in Figure 13a. In the physical experimental setup, the system operates around the 16-channel ACCUSPECT M ECT instrument (Accuspect Technology, Derby, UK). During the experiment, a heating belt was used to heat the Inconel 718 alloy specimen containing grooves of varying depths, while a chiller system ensured the stable operation of the probe in the high-temperature environment. By acquiring the voltage signals from different defects using this platform, the high-temperature detection capability of the optimized probe was systematically evaluated.

A flat plate specimen, measuring 91 mm in length, 88 mm in width, and 2 mm in thickness, was fabricated from Inconel 718 nickel-based superalloy. To simulate realistic microcracks, artificial rectangular notches with a constant width of 0.2 mm were introduced onto the specimen surface via electrical discharge machining. The geometric parameters, including the specific depths and spatial distribution of these notches, are illustrated in Figure 13b. To validate the effectiveness of the preceding probe parameter optimization, comparative inspections were initially performed at ambient temperature to evaluate the performance difference before and after optimization. Subsequently, the specimen was heated to the target temperature to conduct elevated-temperature microcrack inspections, thereby verifying the high-temperature service capability of the optimized probe.

### 4.2. Fabrication of the High-Temperature Absolute Eddy Current Probe

A custom absolute eddy current probe was fabricated for high-temperature crack detection (Figure 13c). The assembly comprises an excitation coil, a detection coil, a ferrite core, a ceramic tube, a copper shield, and a 304 stainless steel casing. To enhance sensitivity, the ferrite core (1 mm diameter, 8 mm height) concentrates the magnetic flux [21]. Using the optimized parameters, both coils were wound directly onto the core with 0.08 mm enameled copper wire. Each coil consists of 200 turns (4 layers of 50 turns), separated by a 0.4 mm gap.

For high-temperature environments, a ceramic tube thermally and electrically isolates the internal components from the casing. A copper layer is also included to shield against external electromagnetic interference. Furthermore, the casing integrates a continuous water-cooling system. The circulating water cools the probe, maintaining stability for accurate inspections.

### 4.3. Probe Performance Comparison at Ambient Temperature

**Figure 14:**
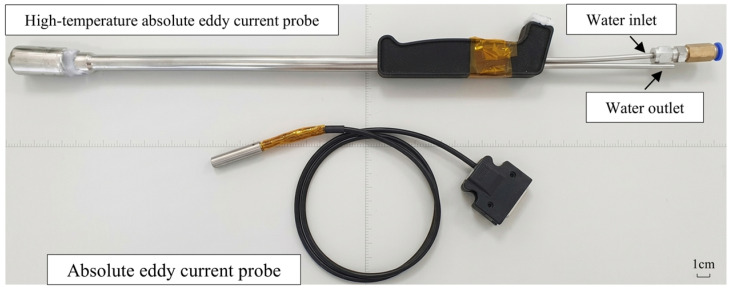
Photographs of the absolute eddy current probes: (**top**) developed water-cooled high-temperature probe; (**bottom**) conventional ambient probe.

To rigorously evaluate the detection capability and sensitivity of the optimized high-temperature absolute eddy current probe, a conventional absolute probe was fabricated to serve as a reference (the structural schematic and parametric comparisons are provided in Figure 14 and Table 4).

The coils of this reference probe were similarly wound using enameled copper wire with a diameter of 0.08 mm. The winding consisted of 300 turns in total, arranged in 5 distinct layers with 60 turns per layer. A commercially available ferrite core, measuring 2 mm in diameter and 10 mm in height, was employed, with both coils symmetrically secured at the mid-section of the core. Subsequently, crack inspection experiments were conducted on the aforementioned nickel-based alloy specimen (Figure 13b) utilizing both probe configurations.

**Figure 15:**
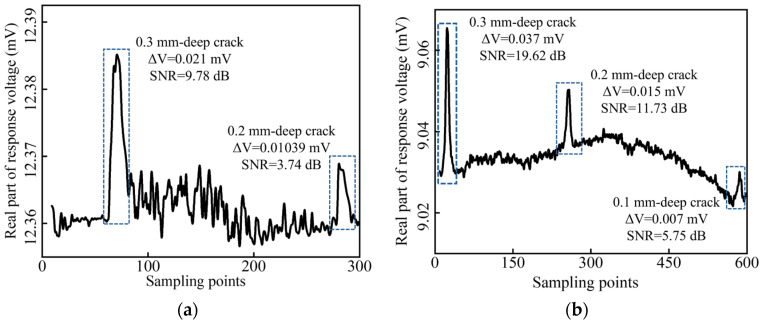
Real part of experimental response voltage for crack detection in Inconel 718 specimen at ambient temperature: (**a**) Conventional absolute probe; (**b**) Developed high-temperature absolute probe.

Figure 15 shows the response voltage amplitude profiles obtained by scanning the notched specimen with both probe configurations at ambient temperature. As shown in Figure 15a, the conventional reference probe exhibits significant baseline wandering and severe background noise during the scan. This probe can identify the larger notches with depths of 0.3 mm and 0.2 mm. However, its overall signal-to-noise ratio (SNR) is poor. The signal at the 0.2 mm-deep crack is only approximately 3.74 dB. Consequently, the signal from the 0.1 mm minute notch is completely obscured by random noise and cannot be effectively extracted.

However, the optimized high-temperature absolute eddy current probe (Figure 15b) demonstrates excellent defect resolution. First, its signal baseline is much smoother. The high-frequency background noise is also significantly suppressed. Second, the detection sensitivity is greatly improved. For instance, for the 0.3 mm-deep crack, experimental results confirm that the optimized probe exhibits superior sensing capabilities. Specifically, the peak response amplitude improved from 0.021 mV to 0.037 mV, and the SNR was enhanced from 9.78 dB to 19.62 dB, demonstrating the robustness and sensitivity of the newly designed probe. Due to the high SNR and improved sensitivity, the optimized probe clearly captures the features of the 0.3 mm and 0.2 mm defects. Furthermore, it can extract the minute signal from the 0.1 mm-deep crack.

These comparative results strongly demonstrate that the optimization of coil parameters and core size based on preliminary simulations is highly successful. This optimization effectively reduces noise levels and significantly improves the overall signal quality. It lays a solid foundation for reliable crack evaluation at high temperatures.

### 4.4. Evaluation of Crack Detection Capability at Elevated Temperatures

After verifying the optimized probe’s sensitivity at ambient temperature, its reliability was further evaluated at elevated temperatures. The specimen was continuously heated from room temperature to 700 °C using a flexible heating tape. The complete experimental setup for these elevated-temperature inspections is illustrated in Figure 13a.

**Figure 16:**
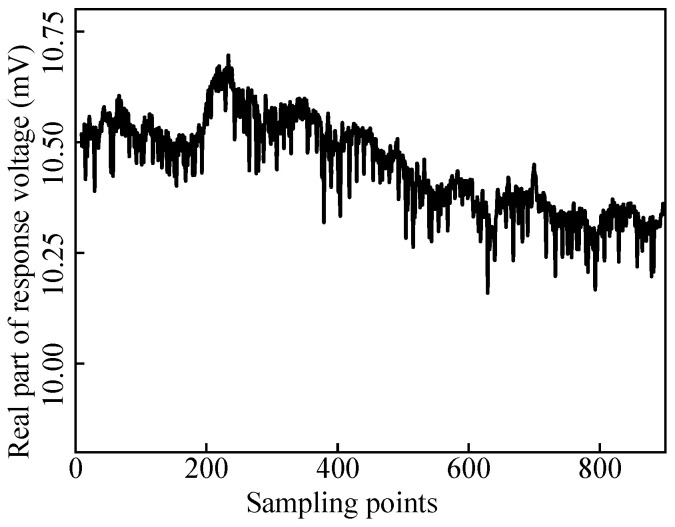
Real part of experimental response voltage for the conventional probe scanning Inconel 718 at 180 °C.

Firstly, the thermal endurance limit of the conventional reference probe was tested. Figure 16 shows its response signal at 180 °C. the signal became completely distorted when the temperature reached 180 °C. This distortion was caused by severe thermionic noise and significant baseline wandering. These effects completely obscured any valuable defect features. This proves that the conventional probe cannot operate in high-temperature environments without thermal protection. Therefore, all subsequent tests only used the water-cooled optimized probe.

**Figure 17:**
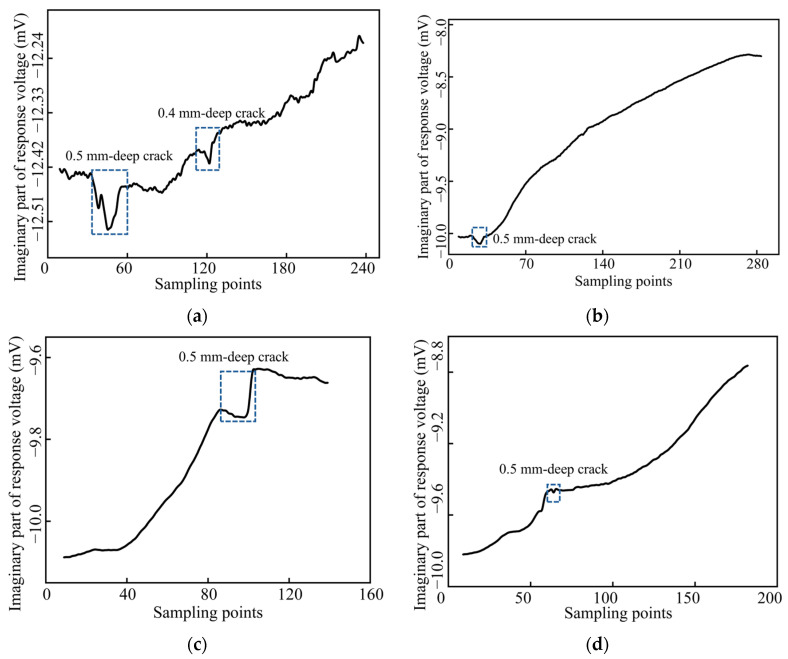

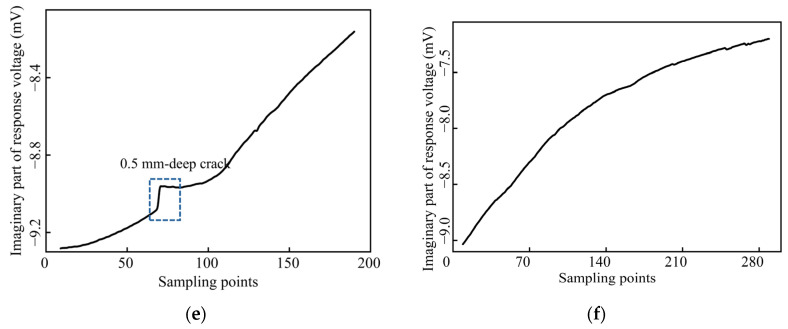
Imaginary part of experimental response voltage for crack detection in Inconel 718 specimen at different temperatures. (**a**) 400 °C; (**b**) 500 °C; (**c**) 550 °C; (**d**) 600 °C; (**e**) 650 °C; (**f**) 700 °C.

Subsequently, the optimized high-temperature absolute eddy current probe scanned the artificial microcracks at specimen temperatures from 400 °C to 700 °C. Figure 16 illustrates the imaginary part of the dynamic scanning response signals for artificial cracks. As shown in Figure 17, the imaginary part is severely contaminated by baseline drift and noise, rendering the crack features indistinguishable. This degradation is primarily attributed to the dynamic lift-off effect during scanning. The actual lift-off distance fluctuates continuously due to probe thermal expansion and water-cooling vibrations. Because the inductance-dominated imaginary part is extremely sensitive to these geometric fluctuations, the weak crack signals are completely overwhelmed. Conversely, the real part represents the Joule heating losses within the material. It demonstrates strong stability against thermal drift, clearly resolving the 0.3~0.5 mm cracks. Therefore, to ensure maximum detection reliability, the real part was selected as the primary indicator for performance evaluation.

**Figure 18:**
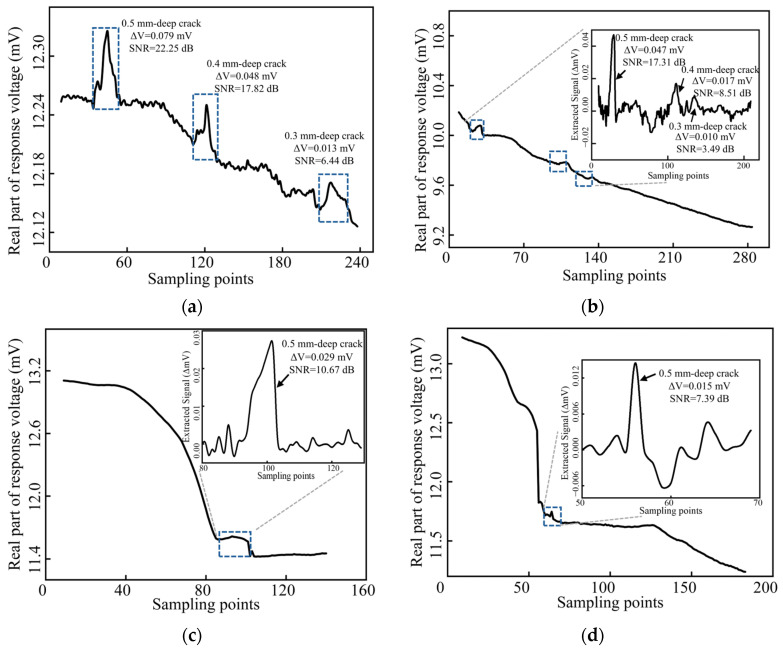

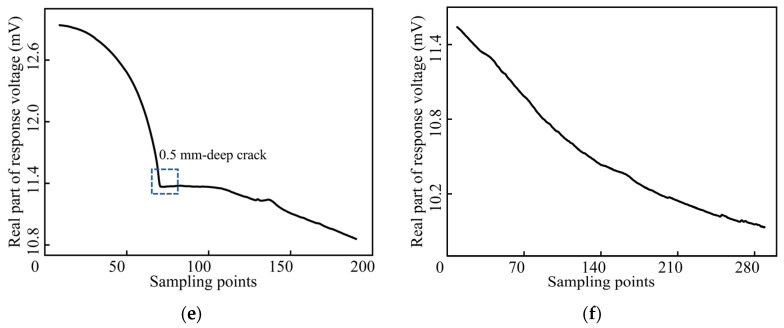
Real part of experimental response voltage for microcrack detection in Inconel 718 specimen at different temperatures. (**a**) 400 °C; (**b**) 500 °C; (**c**) 550 °C; (**d**) 600 °C; (**e**) 650 °C; (**f**) 700 °C.

Figure 18 displays the real part of the defect response signals extracted after background separation. The internal water-cooling system effectively blocks heat conduction. It successfully keeps the working temperature of the Mn-Zn ferrite core below its Curie point. This thermal protection prevents the probe from demagnetizing and degrading. At 400 °C, the real part clearly identifies all micro-cracks. The 0.5 mm crack yields an exceptional SNR of 22.25 dB. The 0.4 mm crack yields 17.82 dB. Even the shallowest 0.3 mm crack remains distinguishable with an SNR of 6.44 dB.

However, the signal amplitude inevitably decreases as the specimen temperature rises. The core physical mechanism relies on the temperature evolution of the material properties. The relative permeability of the Inconel 718 alloy shows no obvious fluctuation. Instead, the rising temperature triggers severe thermal vibration of the crystal lattice. This enhanced phonon scattering severely hinders electron migration. Consequently, the electrical conductivity of the alloy drops significantly [22]. This reduction increases the eddy current skin depth. It also weakens the surface-induced current density. Therefore, the defect signals undergo a natural attenuation. Despite this attenuation, the 0.5 mm crack is successfully captured up to 650 °C. It maintains a reliable SNR of 7.39 dB at 600 °C. Even at 650 °C, the 0.5 mm crack profile remains identifiable. The signal finally vanishes into the massive thermal noise at 700 °C. These experimental results perfectly confirm the simulation trends.

Therefore, the real component of the eddy current signal is selected as the primary feature for high-temperature crack characterization due to its superior immunity to thermal drift. While the real component successfully preserves distinct defect profiles and SNR up to 650 °C, the imaginary component exhibits extreme instability under intense thermal perturbations. The synergistic effects of elevated temperatures and cooling-induced vibrations induce severe baseline distortion along the imaginary axis. This pronounced drift inevitably obscures low-amplitude defect signatures, escalating the probability of false negatives. Therefore, utilizing the real component as the core evaluation metric guarantees robust, high-fidelity flaw detection under high-temperature conditions.

To accurately evaluate these attenuated defect features amidst the high-temperature thermal drift, a baseline correction was applied to the raw signals at 500 °C, 550 °C, and 600 °C. The crack signals extracted after this calibration are displayed in the inset plots of Figure 18b–d. As shown in the insets, the 0.3 mm-deep crack could be clearly resolved at 500 °C. Furthermore, even at the extreme temperature of 650 °C (Figure 18e), the probe could still successfully identify the 0.5 mm-deep crack.

To further quantify the probe’s reliability and robustness in extreme heat, we performed five independent dynamic scans for cracks of varying depths at each temperature. We extracted the maximum real voltage variation for each scan to calculate the mean and standard deviation (SD). The calculation of the coefficient of variation (CV) is as follows:

(7) $$\gamma =\frac{\alpha }{\beta }\times 100\%$$

where α represents the standard deviation and β is the mean.

**Figure 19:**
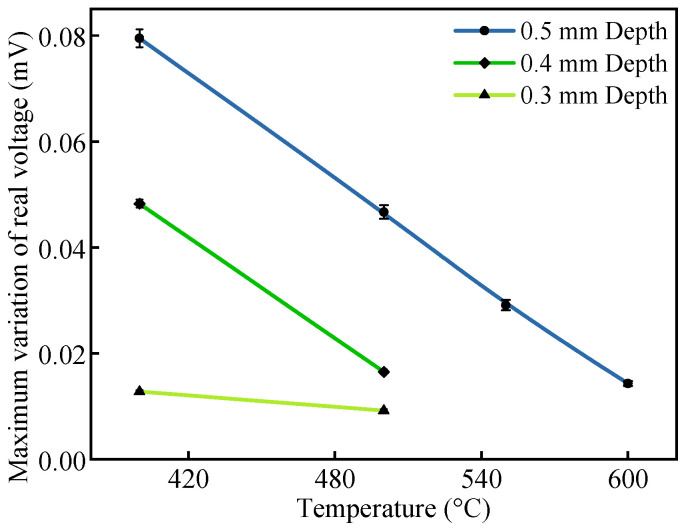
Peak variation of the real voltage component as a function of temperature for various crack depths in Inconel 718. Error bars denote the standard deviation of five independent measurements.

To ensure an accurate and fair comparison of signal stability at different temperatures, we introduced the CV as defined in Equation (7). This serves as the core metric to measure the system’s resistance to thermal noise. Figure 19 and Table 5 detail the mean attenuation trends and error distributions for all signal groups.

As shown in Table 5, although the maximum real voltage attenuates significantly with increasing temperature, the CV for all signal groups remains at exceptionally low levels. Taking the 0.5 mm crack as an example, even at the high temperature of 600 °C, its CV is only 3.5%. Under dynamic eddy current scanning conditions, the coefficients of variation for all signal groups are below 5%. This low data dispersion objectively demonstrates that the optimized water-cooled probe possesses good signal reproducibility and system robustness in thermal environments.

On the other hand, the data also clearly reveals the detection limits of the probe at different temperatures. As the specimen temperature rises to 550 °C and above, the weak characteristic signals of the 0.4 mm and 0.3 mm cracks are completely submerged by the thermal background noise, making effective extraction impossible (as indicated by “/” in Table 5). By combining the statistical results from Figure 18 and Table 5, the effective detection range of the probe can be clearly defined: within the medium-to-high temperature range of 400 °C to 500 °C, the probe achieves high-sensitivity identification of the 0.3 mm crack; meanwhile, in thermal environments up to 600 °C, it retains the reliable capability to stably extract features from the 0.5 mm crack.

It is worth noting that as the testing temperature further increases to 650 °C, the severe baseline drift caused by the evolution of the alloy’s high-temperature properties makes it difficult to accurately extract the weak response voltage amplitude induced by the defects. Consequently, statistical analyses such as the CV cannot be performed. However, as shown in Figure 18e, a local characteristic perturbation and a distinct signal inflection point induced by the 0.5 mm crack can still be clearly observed on the steep dynamic scanning baseline curve. This fully demonstrates that even in the extreme thermal environment of 650 °C, although the optimized probe has exceeded the boundary for stable amplitude feature extraction, it still possesses the capability for qualitative identification of the 0.5 mm deep crack.

## 5. Conclusions

This study proposed and validated a water-cooled absolute eddy current probe for high-temperature microcrack detection in Inconel 718. Based on Multiphysics simulations and dynamic scanning experiments, the main conclusions are as follows:

1. Optimizing the coil and magnetic core significantly improved the probe’s sensitivity over conventional absolute designs. For a 0.3 mm deep crack, the peak amplitude increased by 76.2% (from 0.021 mV to 0.037 mV), and the SNR improved by nearly 10 dB (from 9.78 dB to 19.62 dB).
2. At 650 °C, the water-cooling system maintains the magnetic core below its Curie point, preventing demagnetization. Consequently, the high-temperature signal attenuation is not a sensor artifact, but stems from the alloy’s reduced electrical conductivity and the corresponding increase in skin depth.
3. The probe exhibits outstanding resistance to thermal noise interference. In the temperature range of 400 °C to 500 °C, it achieves high-sensitivity identification of 0.3 mm cracks at 600 °C, it enables reliable detection of 0.5 mm cracks with a low CV ≤ 3.5%; under the extreme condition of 650 °C, it still retains the capability for qualitative identification.

## Figures and Tables

**Table 1:** Physical property parameters of materials used in the simulation.

Geometric Model	Material	Electrical Conductivity (S/m)	Relative Permeability
Specimen	Inconel 718	9.22 × 10^5^	1
Air	/	/	1
Core	Mn-Zn ferrite	0.167	4 × 10^3^
Coil	Copper	5.998 × 10^7^	1

**Table 2:** Simulation results with different meshes.

Mesh Size (mm)	Probe Position L (mm)	Real Part of Response Voltage (V)	Amplitude Error (%)
0.03	0	−2.1485	0
0.04	0	−2.1485	0
0.05	0	−2.1487	0.0093
0.06	0	−2.1489	0.0186
0.08	0	−2.1492	0.0326

**Table 3:** Physical properties of copper and Inconel 718 at different temperatures.

Temperature (°C)	20	400	500	600
Conductivity of copper (MS/m)	5.96	2.22	1.9	1.66
Young’s modulus of copper (GPa)	131.4	106.7	98.7	90.6
Conductivity of Inconel 718 (MS/m)	0.0922	0.0775	0.0769	0.0758
Young’s modulus of Inconel 718 (GPa)	200	180	174.4	168.2

**Table 4:** Comparison of probe parameters before and after optimization.

Parameters	Absolute	High-Temperature Absolute
Wire diameter (mm)	0.08	0.08
Number of turns	300	200
Core height (mm)	10	8
Core diameter (mm)	2	1

**Table 5:** Statistical results of the maximum real voltage variation for microcracks of different depths at varying testing temperatures.

Temperature (°C)	0.5 mm Mean (mV)	0.5 mm SD	0.5 mm CV	0.4 mm Mean (mV)	0.4 mm SD	0.4 mm CV	0.3 mm Mean (mV)	0.3 mm SD	0.3 mm CV
400	0.07947	0.00168	2.1%	0.04826	0.00077	1.6%	0.01278	0.00027	2.1%
500	0.04669	0.00128	2.7%	0.01649	0.00046	2.8%	0.00918	0.00027	2.9%
550	0.02911	0.00098	3.4%	/	/	/	/	/	/
600	0.01430	0.00050	3.5%	/	/	/	/	/	/

## Data Availability

All data included in this study are available upon request via contacting the corresponding author.
